# A poetic story: lessons learnt from the world's largest breast cancer window of opportunity study

**DOI:** 10.1186/1745-6215-16-S2-P83

**Published:** 2015-11-16

**Authors:** Judith Bliss, Alexa Gillman, Lucy Kilburn, James Morden, Kally Sidhu, Maggie Wilcox, Abigail Evans, Christopher Holcombe, Kieran Horgan, Anthony Skene, Raghavan Vidya, John Robertson, Mitch Dowsett, Ian Smith

**Affiliations:** 1 Clinical Trials and Statistics Unit, The Institute of Cancer Research, London, UK; 2 Royal Marsden Hospital, London, UK; 3 Independent Cancer Patients’ Voice, London, UK; 4 Poole Hospital, Poole, UK; 5 Royal Liverpool University Hospital, Liverpool, UK; 6 Leeds General Infirmary, Leeds, UK; 7 Royal Bournemouth Hospital, Bournemouth, UK; 8 Stafford Hospital, Stafford, UK; 9 Nottingham University Hospitals, Nottingham, UK

## 

Hormone sensitive breast cancer (BC) is a common disease in postmenopausal women. Generally seen as less aggressive than other BC subtypes patients have a continued risk of relapse for 15+ years (EBCTCG, 2011) thus the cumulative risk is not insubstantial. Efforts continue to identify patients who are at continued residual risk of relapse in order to develop new treatment strategies. Previous trials (IMPACT, 2007) suggested that aromatase inhibitor (AI) treatment for 2 weeks in the peri-surgical *window-of-opportunity* results in detectable biomarker changes (Ki67) and predicts long term outcome. Gene expression profiling offers opportunities to identify patients demonstrating early resistance to endocrine therapy.

POETIC was a UK-wide RCT devised to provide definitive results on the role of perioperative (AI) treatment. Biopsies were taken at diagnosis and 2 weeks later at surgery thus allowing an in vivo assessment of AI sensitivity. To succeed, POETIC needed to overcome substantial barriers in relation to compliance with cancer wait times, recruitment of women at diagnosis, varied clinical practice and to ensure receipt of sufficient quality tissue samples for analysis of biomarker endpoints. Patient advocates were involved from inception.

POETIC succeeded in recruiting 4486 women from 130 UK centres. Paired tissue samples were received for 96% patients. Lessons learnt in rolling out the world's largest window-of-opportunity BC trial illustrate the viability and potential of this experimental model as a vehicle for testing early biological effects of novel agents and to identify women most likely to be at long term residual risk of relapse.

